# Discrimination of plant root zone water status in greenhouse production based on phenotyping and machine learning techniques

**DOI:** 10.1038/s41598-017-08235-z

**Published:** 2017-08-15

**Authors:** Doudou Guo, Jiaxiang Juan, Liying Chang, Jingjin Zhang, Danfeng Huang

**Affiliations:** School of agriculture and biology, Shanghai Jiao Tong University, Shanghai, People’s Republic of China

## Abstract

Plant-based sensing on water stress can provide sensitive and direct reference for precision irrigation system in greenhouse. However, plant information acquisition, interpretation, and systematical application remain insufficient. This study developed a discrimination method for plant root zone water status in greenhouse by integrating phenotyping and machine learning techniques. Pakchoi plants were used and treated by three root zone moisture levels, 40%, 60%, and 80% relative water content. Three classification models, Random Forest (RF), Neural Network (NN), and Support Vector Machine (SVM) were developed and validated in different scenarios with overall accuracy over 90% for all. SVM model had the highest value, but it required the longest training time. All models had accuracy over 85% in all scenarios, and more stable performance was observed in RF model. Simplified SVM model developed by the top five most contributing traits had the largest accuracy reduction as 29.5%, while simplified RF and NN model still maintained approximately 80%. For real case application, factors such as operation cost, precision requirement, and system reaction time should be synthetically considered in model selection. Our work shows it is promising to discriminate plant root zone water status by implementing phenotyping and machine learning techniques for precision irrigation management.

## Introduction

Over the past decade, the environmental information based methods for precision management have been widely applied in greenhouse practice. The ambient climate of greenhouse such as temperature, humidity, and root zone water content is monitored closely as the reference for crop management^1^. Water and fertilizer are the most important inputs for greenhouse crop because the plant growth is significantly affected by plant water and nutrition status. Root zone water status has a significant influence on plant evapotranspiration rate^2^. Root zone water status indicates the moisture situation of plant root growth environment and highly correlates to plant growing status^3^. Monitoring of root zone water status could help to establish the precision management strategy on irrigation in crop production. However, conventional probe sensors for moisture measurement are limited on detecting the actual substrate water status effectively in trays. Several commercialized soil moisture sensors such as tensiometer, neutron probes and time domain reflectometry probes have been widely used in pot planting but not applicable for seedlings mainly because the high costs, unsuitable size, and unreliable measurements. The volume of cell in plug tray is minimal resulting that common probe sensors can hardly insert into substrates. In addition, due to the uneven water distribution among different plug trays, the growers require continuous and real-time water content monitoring for large -scale rather than for single point, to prevent the seedlings from water stress and growth restriction. The relationship between plug tray weight and root zone water content was studied to provide a weighting based irrigation control strategy^4^. However, irrigation based on tray weight is labor intensive and inaccuracy in practice.

With the development of sensing technology, real-time monitoring of physiological and ecological information of the plant itself provides an approach to indicate root zone water status^5, 6^. Plant physiological traits such as tissue water status, stomatal conductance, and sap flow are known sensitively responding to water stress. The status of plant water content could be a useful indicator for irrigation^7^. Phenotypic traits such as leaf area, leaf angle, and thermal temperature show strong correlations with the root zone water status^8^. However, most of the plant -based irrigation methods are still at research/developing stage and little used yet for practice (except for thermal sensing in some situations). Systematical research on phenotypic traits selection for irrigation has seldom been reported.

Rapid and non-destructive plant sensing method is needed for plant-based information acquisition. The application of machine vision technology could be the solution. Plant phenotypic traits can be quickly and efficiently obtained by imaging-based automatic inspection and analysis^5^. Therefore, machine vision has the potential to realize the rapid, reliable, real-time monitoring of plant status with low cost, which meets the requirements for precision irrigation scheduling^9^. Machine vision technology has been applied in plant monitoring such as the detection of plant abiotic stress through color variation, plant movement monitoring, textural features detection, canopy temperature measurement and detection of plant biotic disease^8, 10–13^. With the development of phenotyping technology, various imaging sensors have been used to collect phenotypic parameters which are related to plant stress in automated or semi-automated high-throughput plant phenotyping platforms^14, 15^. However, data generated by these platforms has enormous volume, variety, velocity, and veracity, which must be efficiently analyzed. The classical statistical method is difficult to analyze the high-dimensional and dynamical image data, resulting limited phenotyping interpretation^16^.

Machine learning, a type of artificial intelligence has good performance in efficiently dealing with large amount of data in various domains. It is promising in phenotyping applications on features extraction and patterns identification from large dataset through holistic approaches. Machine learning algorithm has few input factors with high predict accuracy and strong adaptability. Several machine learning algorithms, such as support vector machine (SVM), artificial neural network (ANN) and random forest (RF), have been used for classification model development^17, 18^.

In practice, machine learning method has been applied to remote sensing system and irrigation system^19, 20^. Many studies have reported plant stress phenotyping using machine learning in identification, classification, quantification, and prediction of plant water status, but few focused on phenotyping root zone water stress^10, 21, 22^. It is crucial to detect plant water stress at the early stage before any damage observed with visible wilting, which affect subsequent plant growth and quality. Correlating root zone water status with plant phenotype to supervise irrigation system could be a practical way in precision irrigation system with easy operation and quick response.

Pakchoi (*Brassica campestris ssp*. L*. chinensis*) is one of typical vegetables in greenhouse production, which is sensitive to water status. During the past few years, commercial production of leafy vegetable has been initiated and developed both in the greenhouse (solar plant factory) and artificial light plant factory^23^. Growing baby pakchoi with plug trays using substrate in greenhouse is a promising production mode because it is suitable for mechanized harvesting and precision management, which improves the production efficiency^24^. The accurate irrigation scheduling is critical for safe and high quality of greenhouse vegetable, as well as improving the resource utilization and production profits. This study aims to discriminate plant root zone water status nondestructively, taking pakchoi as the plant material, which is a part of on-going research on developing machine vision-based precision irrigation system for factory production of pakchoi. The objectives of this study are: (1) investigating the relationship between root zone water status and plant growth; (2) developing a root zone water status discrimination model by applying phenotyping technology and machine learning algorithms; (3) evaluating the performance of developed models in various application scenarios.

## Results and Discussion

### The effect of root zone water status on plant growth

**Figure 1:**
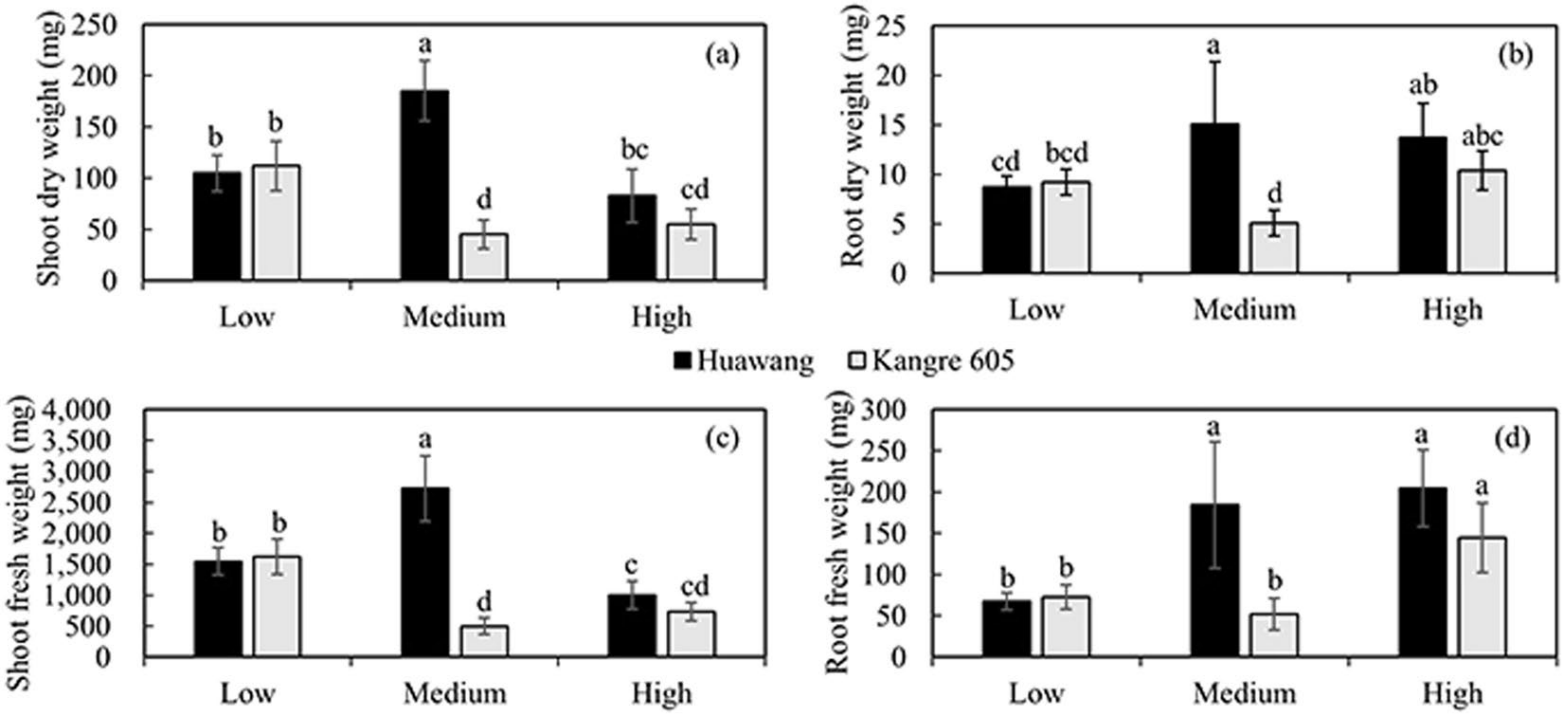
Effects of different water treatments on the growth of two pakchoi cultivars. (**a**) Shoot dry weight per plant under three different root zone water content treatments, 40% (low), 60% (medium), and 80% (high) relevant water content, (**b**) root dry weight per plant under three treatments, (**c**) shoot fresh weight under three treatments, and (**d**) root fresh weight per plant under three treatments. Means with the same letter are not significantly different (P > 0.01). One half-bar represents the standard deviation.

The effect of root zone water status on pakchoi plant growth was investigated under three relative water content treatments, 40% (low), 60% (medium), and 80% (high). The fresh and dry weight of shoot and root for both cultivars were significantly different between treatments as shown in Fig. 1 with ANOVA test (p < 0.01). Treatments had different effects between cultivars. For cultivar “Huawang”, treatment of medium water content had the highest values of fresh and dry weight of shoot and root (fresh weight as 2727.1 mg and 184.4 mg for shoot and root respectively, and dry weight as 185.1 mg and 15.1 mg per plant for shoot and root respectively), while cultivar “Kangre 605” had the lowest values under such treatment (fresh weight as 500.3 mg and 52.0 mg for shoot and root respectively, and dry weight as 45.3 mg and 5.1 mg per plant for shoot and root respectively). The differences of pakchoi growth were significant between two cultivars under the treatment of medium water content (p < 0.01 for all comparisons), but there were no significant differences observed between cultivars under the other two treatments. This is mainly because “Kangre 605” is a heat-resistant cultivar, which has the ability of growing well under water stress conditions. “Huawang” prefers moderate water condition, and both the excess and deficiency of water supply could affect the accumulation of biomass.

### Relationship between root-zone water status and phenotypic traits

#### Phenotypic traits extraction

Two types of images were acquired, visible and near-infrared (NIR) image (Supplementary Fig. S1). There were in total 37 phenotypic traits extracted from images for each plant (Table 1). These phenotypic traits were classified into three categories for easy description, morphological trait, color trait, and NIR trait, including 19, 6, and 12 traits respectively. Morphological traits mainly indicated the shape feature of target plant region, such as circumference and eccentricity. Color traits were generated from color images, including the pixel value information of R (red), G (green), and B (blue) components. NIR traits were the pixel intensity information of different ranges in NIR images.

**Table 1:** Phenotypic traits extracted from images for each plant.

No.	Trait name	Description	Category
1	NIR_area	Area of near-infrared plant (mm^2^)	NIR
2	1 A	Ratio of plant pixels with NIR intensity in 170–186	NIR
3	1 R	Number of plant pixels with NIR intensity in 186–202	NIR
4	2 A	Ratio of plant pixels with NIR intensity in 186–202	NIR
5	2 R	Number of plant pixels with NIR intensity in 202–218	NIR
6	3 A	Ratio of plant pixels with NIR intensity in 202–218	NIR
7	3 R	Number of plant pixels with NIR intensity in 218–234	NIR
8	4 A	Ratio of plant pixels with NIR intensity in 218–234	NIR
9	4 R	Number of plant pixels with NIR intensity in 234–250	NIR
10	5 A	Ratio of plant pixels with NIR intensity in 234–250	NIR
11	5 R	Average intensity of near-infrared plant pixels	NIR
12	NIR intensity	Number of plant pixels with NIR intensity in 170–186	NIR
13	Mincirclediam	Min Enclosing Circle Diameter (mm)	Morphological
14	Normsmallpax	2nd Moment Principle Axis Small Norm	Morphological
15	Normlargrpax	2nd Moment Principle Axis Large Norm	Morphological
16	Minrectarea	Min Area Rectangle Area (mm^2^)	Morphological
17	Mindistcenbdy	Center Of Mass To Boundary Distance (mm)	Morphological
18	Vrectsizey	Height of the smallest vertical rectangle covering the plant (mm)	Morphological
19	Vrectsizex	Width of the smallest vertical rectangle covering the plant (mm)	Morphological
20	Compactness	Square of the objects perimeter to object area	Morphological
21	Real area	Area of plant (mm^2^)	Morphological
22	Paxratio	2nd Moments Principal Axis Ratio	Morphological
23	Circumference	Perimeter of plant excluding holes (mm)	Morphological
24	Eccentricity	The ratio of the distance between the foci to the length of the major axis	Morphological
25	Maxdiam	Maximum distance between two points on the plant boundary (mm)	Morphological
26	Roundness	The ratio between the inscribed and the circumscribed circles	Morphological
27	Bdryround	Boundary Point Roundness	Morphological
28	Bdrycount	Boundary Point Count	Morphological
29	Bdrytoarearatio	Boundary Points To Area Ratio	Morphological
30	Conhullcirc	Convex Hull Circumference (mm)	Morphological
31	Conhullarea	Convex Hull Area (mm^2^)	Morphological
32	Mean Color Blue	Average color in Blue range of the RGB color space	Color
33	Mean Color Blue Variance	The variance of average color in Blue range of the RGB color space	Color
34	Mean Color Green	Average color in Green range of the RGB color space	Color
35	Mean Color Green Variance	The variance of average color in Green range of the RGB color space	Color
36	Mean Color Red	Average color in Red range of the RGB color space	Color
37	Mean Color Red Variance	The variance of average color in Red range of the RGB color space	Color

### Water related phenotypic traits selection

**Figure 2:**
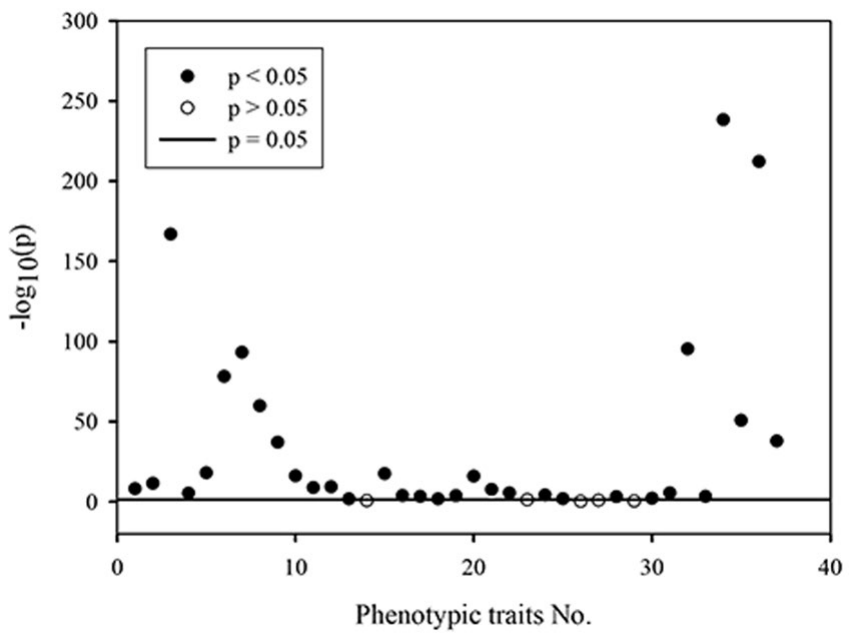
Significance analysis of phenotypic traits with p value threshold as 0.05. The solid horizontal line represents p = 0.05, i.e. −log_10_(p) = 1.301.

In order to increase the efficiency of model development, traits selection is necessary to identify redundant traits which are irrelevant to root zone water status. The significance of 37 extracted phenotypic traits on classifying root zone water status were investigated by using ANOVA (Fig. 2). For better illustration, the p value was transformed into -log10 (p). There were five traits with p > 0.05 (i.e. −log10 (p) < 1.301) including Normsmallpax, Circumference, Roundness, Bdrycount and Bdrytoarearatio (numbered as 14, 23, 26, 28, and 29 in Table 1 respectively). Therefore, the five traits were excluded from the datasets in the following modeling process.

### Root zone water status discrimination model development and validation

#### Modeling potentiality assessment

**Figure 3:**
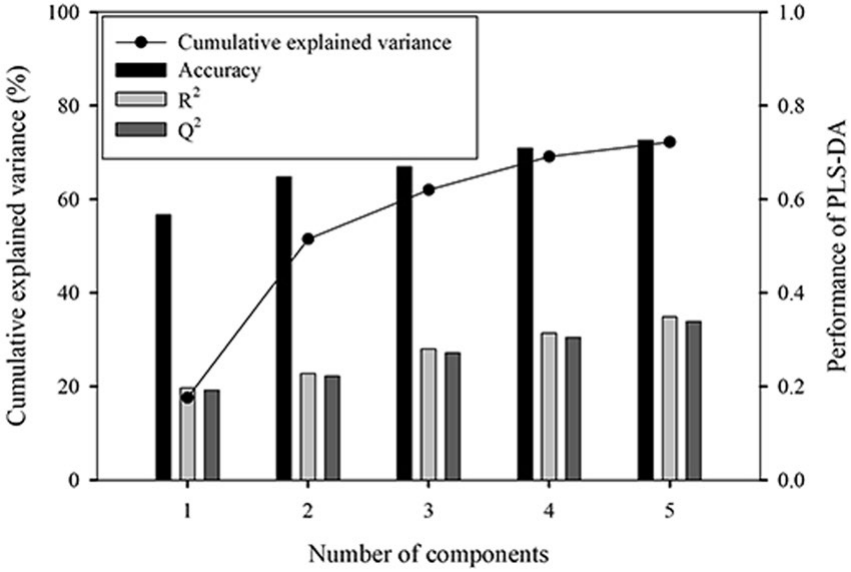
PLS-DA model performance and cumulative variance explained by different number of the top five components.

The Partial Least Squares - Discriminant Analysis (PLS-DA) was conducted to present a visual space distribution of three treatment groups for assessing the modeling potentiality using the 32 selected phenotypic traits. Figure 3 shows the PLS-DA model performance and cumulative variance explained by the top five components. The goodness of fit was quantified by R^2^ while the predictive ability was indicated by Q^2^. The accuracy, R^2^, and Q^2^ of PLS-DA model by using all the top five components reached 73%, 0.35, and 0.34, respectively. The value of R^2^ and Q^2^ should be greater than 0.5 to be taken as an indicator of model acceptability. The cumulative explained variance ranged from 17.6% to 72.2% by the number of components from one to five, with the first three components explaining 66.9% variance. The performance of PLS-DA model improved (R^2^ = 0.67, Q^2^ = 0.66) by excluding the medium root zone water group as the difference between medium water group and the other two groups was not significant. Overall, PLS-DA shows the potential of root zone water status discrimination by phenotypic traits.

#### Discrimination model development

The total dataset used for model development was 2120 samples with 32 traits of each (2120*32), and the dataset was separated into 80% training dataset and 20% testing dataset. The training data set was used for model development. Parameter values for the three modeling algorithms, which are Random Forest (RF), Neural Network (NN), and Support Vector Machine (SVM), were selected by using 10-fold cross validation in three repetitions tuning (Table 2). Among the training dataset, 10% of the data were used as validation in each cross validation. Table 2 also lists the package, function, and program training time of modeling algorithms. All the R codes ran in Rstudio 1.0.44 on the platform of windows 10 × 64 system with an Intel Core i7-5500U CPU and 4 GB RAM.

**Table 2:** Parameter selection result for cross validation training in three modeling algorithms.

Modeling algorithm	Parameter	Parameter range	Parameter selected	R package	Function	Training time (s)
RF	mtry	2, 5, 8	2	randomForest	randomForest()	715.38
	ntree	500	500			
NN	decay	0, 0.1, 0.001	0.1	nnet	nn()	544.37
	size	2, 5, 9	9			
SVM	sigma	10^(−3:0)^	0.1	e1071	svm()	1245.94
	c	10^(1:3)^	1000			

Note: RF - Random Forest, NN - Neural Network, SVM - Support Vector Machine.

Considering the dynamics of the data input and training in practice, the model training time is an important factor to be considered in the optimization process. The training time increases exponentially with the number of parameters and varies substantially between algorithms. Among these three modeling algorithms, NN took the shortest training time as 544.37 seconds, which was approximately 76% of RF and 44% of SVM. The training time consumption of SVM should be considered in real case of decision support system as this method is more sensitive to the data size than others, especially when time is limited and data set is large. The trait contribution analysis in each model could be a way to simplify the model inputs and reduce the training time, which was discussed in section 3.3.

**Figure 4:**
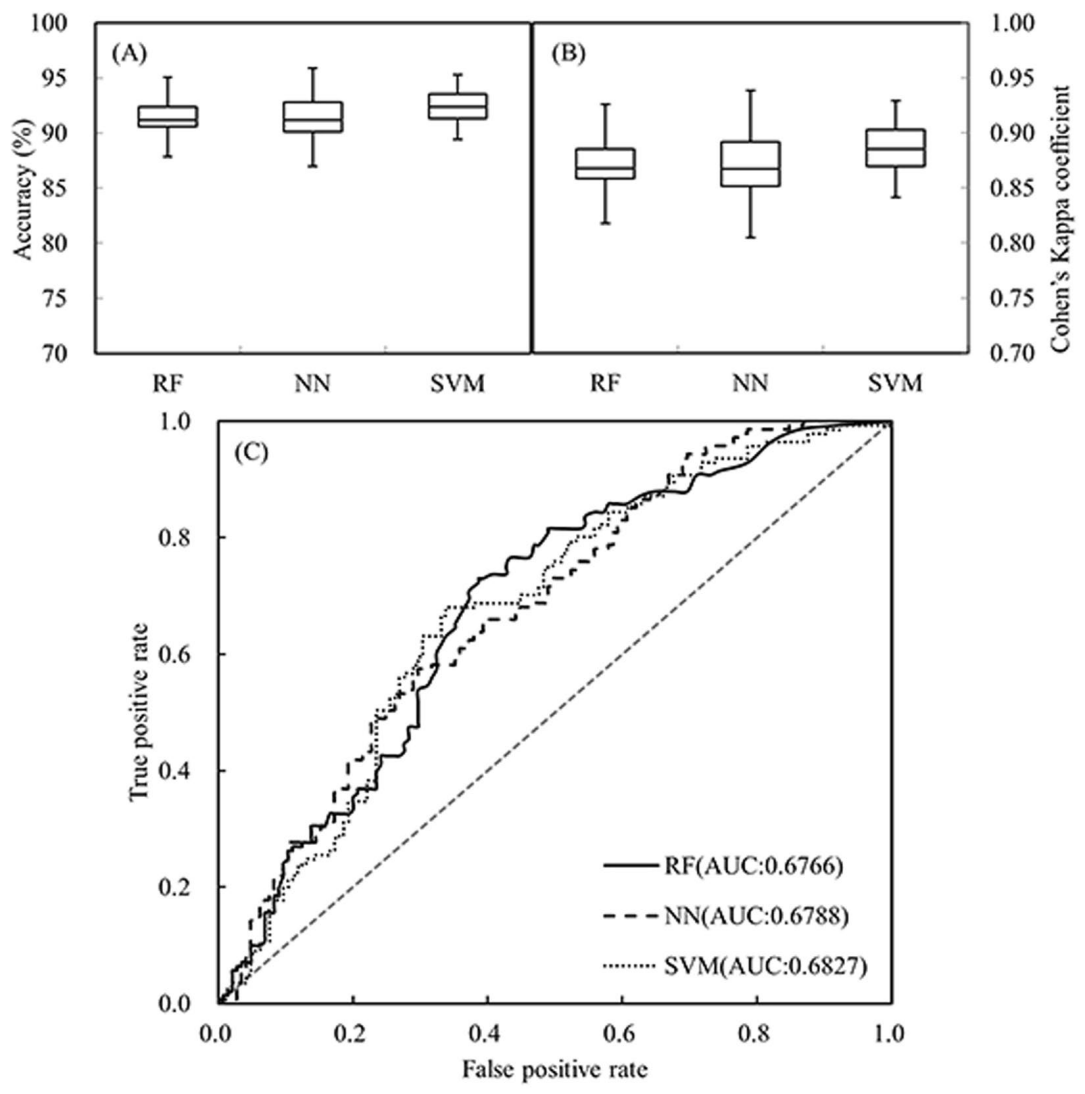
Evaluation result for different models. (**A**) Box-whisker plot of accuracy, (**B**) box-whisker plot of Kappa, and (**C**) the ROC curve of the three developed models. The straight dotted line in subplot (**C**) represents 0.5 AUC.

The mean prediction accuracy and Cohen’s Kappa coefficient (k) of all the three models were higher than 90% and 0.85 respectively, as shown in Fig. 4A,B. SVM model had the best performance with higher accuracy and k value (92.5% and 0.89 respectively) than the other two models. The result also showed that SVM was slightly more stable with less variation than others.

The ROC (receiver operating characteristic) curve was used to evaluate the performance of modeling classifier. Figure 4C shows the ROC curves of the three models. The x-axis represents false positive rate (FPR), and the y-axis represents true positive rate (TPR). The area under the curve (AUC) indicates classification performance, which is the ability of target model to correctly classify the plants in different root zone water status. SVM model had the highest AUC value as 0.6827 among the three models, while the differences were not substantial compared to 0.6766 and 0.6788 AUC of RF model and NN model respectively.

#### Discrimination model validation

Confusion matrix was implemented to validate the three developed models with 2120*32 dataset. The prediction accuracy of each model from cross validation is the sum of the prediction accuracy on the diagonal in each sub-table of Table 3. All the developed models had good prediction accuracy higher than 90%. SVM model had the highest prediction accuracy as 92.5%, but no substantial difference observed on accuracy among the three models (91.3% and 91.4% for RF model and NN model respectively).

**Table 3:** Confusion matrix of cross validation model results.

Model: Random Forest (RF)		Actual class		
		High	Medium	Low
Predicted class	High	30.3%	1.4%	0.9%
	Medium	2.4%	30.1%	1.6%
	Low	0.3%	2.0%	30.9%
**Accuracy**		91.3%		
Predicted class	High	30.2%	1.6%	1.1%
	Medium	2.3%	30.3%	1.5%
	Low	0.6%	1.6%	30.9%
**Accuracy**		91.4%		
Prediction	High	30.5%	1.5%	0.6%
	Medium	1.8%	30.4%	1.1%
	Low	0.7%	1.7%	31.6%
**Accuracy**		92.5%		

Compared to the other two treatments, the medium root zone water group had the highest percentage of false positive (4.0%, 3.4%, 3.8% for RF, NN, and SVM model) and false negative (3.2%, 2.9% and 3.2% RF, NN, and SVM model) among the three models. It could be explained as that medium level is adjacent to both high and low level, which is less distinguishable comparing to the other two levels.

#### Model evaluation under different conditions

**Figure 5:**
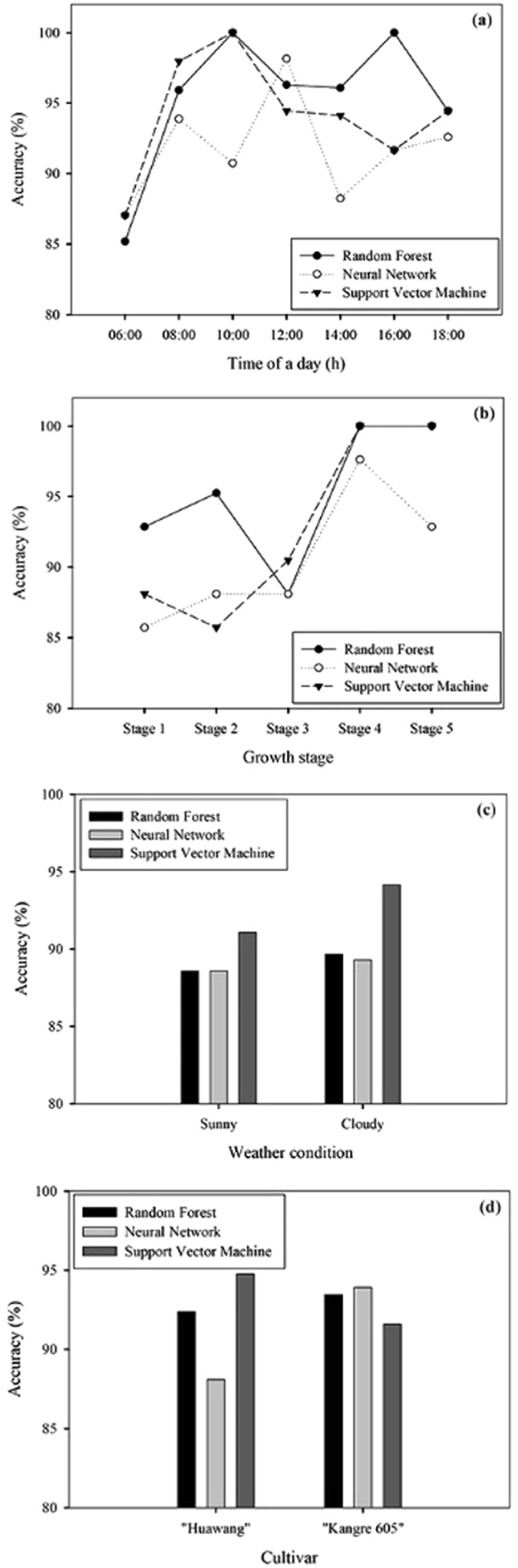
Classification accuracy of developed models in different scenarios. (**a**) Different time of a day, (**b**) growth stage from stage 1 to stage 5, (**c**) weather condition, and (**d**) cultivar.

To investigate the robustness and applicability of the three models in different conditions, the test dataset was reorganized into different scenarios, different time of a day, growth stage (stage 1 to stage 5), weather condition (sunny and cloudy), and cultivar (“Huawang” and “Kangre 605”). The dataset used for each growth was 120*32, the dataset used for each time in day was 240 to 270 records with 32 traits, and the dataset used for each cultivar was 1060*32. All the datasets were separated into 80% training dataset and 20% testing dataset. Figure 5 shows classification accuracy of three models in all scenarios were over 85%. Among the seven time points of a day, the accuracy of the three models were relatively lower at 06:00 h than at other time points (Fig. 5a), which might be caused by the rapid decline of the greenhouse humidity at sunrise resulting in the change of leaf surface moisture status. For NN model, its accuracy dropped at 10:00 h and 14:00 h with the highest accuracy at noon, while RF and SVM model had high accuracy at 10:00 h as 100.0%. For different growth stage, the accuracy of the three models had the trend of increasing (Fig. 5b). The accuracy of RF and SVM model reached 100.0% in the fourth and fifth growth stage which were substantially higher than NN model. In the early stage, leaf area was small resulting in poor discrimination performance. With leaf expanding, the difference of traits between groups increased. In addition, as the treatments of root zone water moisture level was applied during the entire growing period, it would have accumulative effect on plant phenotype, which increased the model classification accuracy in later stages.

The accuracy of the three model were slightly higher in cloudy weather condition than in sunny weather condition (Fig. 5c), which might be because of less environmental fluctuation in cloudy day. SVM model had the highest accuracy in both weather conditions (91.1% and 94.1% respectively). Among the three models, NN model had the largest difference of accuracy between cultivar (5.8%, Fig. 5d) compared to RF and SVM model (1.1% and 3.2% respectively). Overall, RF model performed more stable than the other two considering all the scenarios.

#### Trait contribution analysis in developed models

In order to check the possibility of reducing phenotyping cost and model training time without significant accuracy reduction, sensitivity analysis was conducted to assess the contribution of each trait in developed models. The simplified model with less explanatory variables were developed according to the traits importance evaluation of the model implementation in the root zone water status detection. Table 4 lists the top five of most contributing traits in each model. The explanation of each trait could be referred to Table 1. Different model had different preference on the trait type. In the top five traits, there was no morphological trait in RF model, no NIR trait in NN model, and no color trait in SVM model.

**Table 4:** The top five most contributing traits of each developed model.

Model	Trait name	Contribution (%)	Cumulative contribution (%)	Accuracy reduction (%)
RF	Mean.Color.Green	9.7	32.6	11.6 (from 91.3 to 79.9)
	Mean.Color.Red	9.5		
	1 R	5.6		
	3 R	4.0		
	1 A	3.8		
NN	Mean.Color.Green	6.8	29.9	14.0 (from 91.4 to 77.4)
	Vrectsizeymm	6.3		
	Mean.Color.Blue	6.3		
	Mean.Color.Red	5.8		
	Mean.Color.Green.Variance	4.7		
SVM	4 A	4.8	22.8	29.5 (from 92.5 to 63.0)
	3 A	4.8		
	3 R	4.7		
	4 R	4.6		
	Compactness	3.9		

Overall, color trait and NIR trait appeared more frequently than morphological trait, which shows plant color and NIR characteristics would be more related to root zone water status. This result agrees with several studies in spectroscopy and remote sensing research on the relationship between spectral reflectivity and water related index^13^. The most frequently appearing traits in top five traits of developed models were Mean.Color.Green and Mean.Color.Red. There was only one morphological trait in NN model and in SVM model, named as Vrectsizeymm and Compactness, respectively. This is likely because plant morphological appearance is basically determined by genotype and substantially influenced by environment such as airflow and illumination.

Model classification ability dropped when only the top five traits were used to develop model, named as simplified model. The accuracy of simplified RF model and NN model could still reach 79.7% and 77.4% respectively, while the accuracy of simplified SVM model decreased substantially from 92.5% to 63.0% with 29.5% reduction. For SVM model, the cumulative contribution of the top five traits was 22.8%, lower than that of RF model and NN model (32.6% and 29.9%, respectively), which could be the main reason for the great decrease on the simplified model accuracy. In addition, simplified SVM model did not include color trait, while simplified NN model did not include NIR trait (Table 4), which showed color related traits would better reflect plant root zone water status than the other two trait types in RF and NN models while the SVM model relied more on NIR traits than the others.

## Conclusion

This study developed a root zone water status discrimination method during plant growth by integrating phenotyping and machine learning techniques. Pakchoi plants were used. Three types of phenotyping traits such as morphological trait, color trait, and near-infrared trait were acquired as the inputs for classification models. Three machine learning models were developed, Random Forest (RF), Neural Network (NN), and Support Vector Machine (SVM), with accuracy above 90% for all. The SVM model had the highest accuracy as 92.5% in model development but it took the longest training time as 1245.94 seconds in this study. In addition, the SVM model had the largest accuracy reduction (22.8%) in simplified model developed by the top five most contributing trait resulting from trait contribution evaluation.

The three developed models were evaluated by test dataset (20% of entire data pool) consisting of data from different scenarios, which were different time a day, growth stage, weather condition, and pakchoi cultivar. All the three models reached the overall accuracy higher than 90%. SVM model had the highest value as 92.5%, but no substantial differences were observed among the three. By evaluated in different scenarios, the three models had accuracy over 85% in all scenarios with some fluctuations. The accuracy at the time point of 06:00 h was the lowest for all the three models, and the trend of increasing was observed during the growth of plant. Better performances were observed in cloudy weather condition than in sunny weather condition of the three models. NN model had the largest difference on accuracy between cultivars. Overall, SVM model had the highest classification accuracy, but more stable performance was observed in RF model considering all the scenarios.

This study demonstrates the potential of machine learning approach on discriminating root zone water status based on complex plant phenotyping traits. The developed discrimination method could promote the plant-based irrigation decision making and implementation in practice. Developed models could be further used in precision irrigation system with appropriate modifications if environment or crop change. Further study is suggested to investigate the repaid response of plant phenotypic traits to the change of root zone water status and the performance of classification models on such response. Therefore accumulative effect of water stress on plant growth during the growing period should be minimized with the real-time discrimination to control the potential yield loss. Population phenotyping and water status discrimination are also worth further study due to plant canopy mutual occlusion and growth competition. In addition, phenotyping traits were extracted from images acquired in a constant light environment. Changes in intensity, quality, and beam angle of illumination would have effects on imaging process and hence affect the modeling process. Therefore, research on effective acquisition of phenotyping traits in natural light environment would be a direction to reduce cost and enhance applicability. Our presented work is capable of serving as a basis and supporting for intelligent greenhouse management, especially for irrigation management.

## Materials and Methods

### Plant materials and growth conditions

The experiment was conducted in a commercial greenhouse from May 1^th^ to 29^th^ in year of 2015, located at northeast of Shanghai, China (31°10′ N, 121°36′ E). Two cultivars of pakchoi were used, “Huawang” and “Kangre 605”, under pot substrate cultivation. Commercial substrate (vegetable seedling substrate, Zhongnuo Agriculture Technology Co., Ltd, Huaian, China) composed of peat moss, perlite, and organic matter was used. Plug tray was used at seedling stage. On the tenth day after sowing, seedlings were transplanted to pots (volume of 1.13 L) with one seedling per pot.

The greenhouse air temperature, humidity, photosynthetic photon flux, substrate temperature and electrical conductivity were monitored every 5 minutes by using an automatic data logging system (PM-11 Phytomonitor, Bio Instruments S.R.L., Chisinau, Moldova) with RTH-11 Meter and SMTE sensor. During the whole experimental period, the average air temperature was 24.3 °C with the highest as 36 °C and lowest as 13.7 °C. The relative humidity ranged from 16.4% to 88% with an average of 58%. Mean daily accumulative solar radiation in the greenhouse was 2.62 MJ/(m^2^·d), with a maximum of 5.41 MJ/(m^2^·d) at May 21^st^.

Treatments of root zone moisture level were applied to each pot after transplanting. Root zone moisture was indicated by the relative water content, the percent of volumetric water content comparing to field water capacity. Because of the dynamic change of root zone moisture status, upper limit was applied to define the moisture level of root zone. In other word, irrigation stopped when root zone moisture reached the upper limit. Considering the feasibility of moisture level control and plant growth, three root zone moisture levels were applied, 40%, 60%, and 80% (referred to as ‘Low’, ‘Medium’, and ‘High’ hereafter, respectively). In total, five plants of each cultivar were randomly assigned to each treatment. Irrigation was conducted manually for all plants once a day according to the pot weight.

### Image acquisition and processing

**Figure 6:**
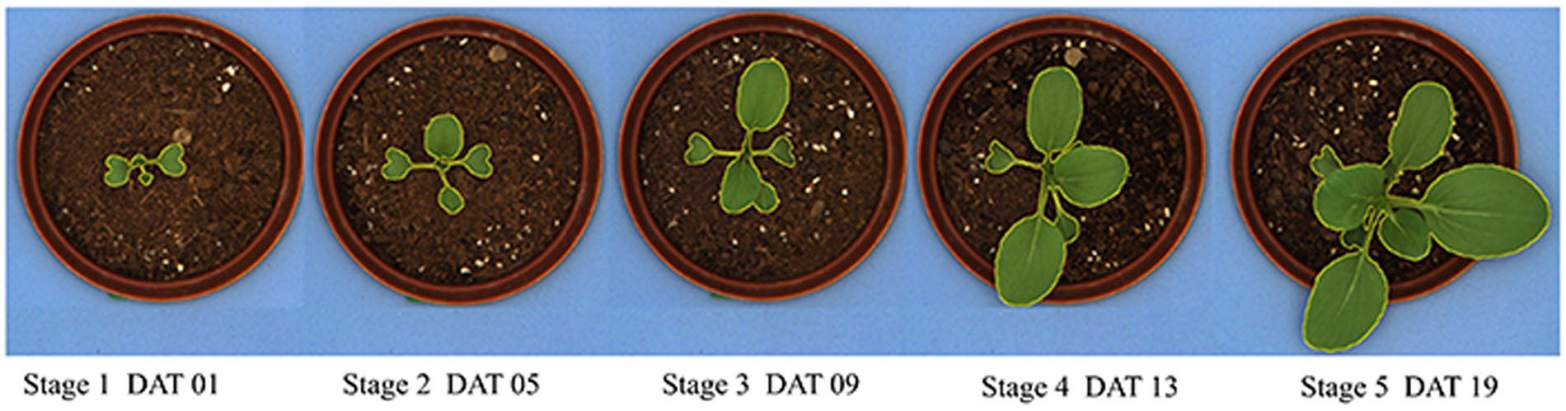
Pakchoi seedling appearance at five growing stages. DAT: days after transplanting.

A commercial phenotyping system (Scanalyzer^3D^, LemnaTec GmbH, Würselen, Germany) was used for image acquisition (Supplementary Fig. S2). Images of each plant were taken daily from top view before irrigation from May 11^th^ to 29^th^, 2015. Two types of image were acquired simultaneously, near-infrared (NIR) and visible (VIS) images. The 20-day growing period was divided into five stages with four days of each, referred to as stage 1, 2, 3, 4, and 5. Plant growing level especially the number of leaves was different at different stage (Fig. 6). The reference growth stage was divided mainly according to the new leaf emerge time under different treatments and adjusted to even distribution for model comparison.

Two image acquisition schedules were applied with one from 06:00 h to 18:00 h at two hour intervals and the other one only at 14:00 h. Weather condition was classified into two scenarios, sunny and cloudy (referred to as ‘S’ and ‘C’ hereafter, respectively) according to weather forecast. Efforts were made to have the two image acquisition schedules for each weather scenario in each stage (Table 5). At the end of 20-day growing period, the fresh and dry weight of each plant sample were measured. Each sample was dried at 105 °C for 1 hour and then at 60 °C to constant weight. The dried sample was weighted to an accuracy of ± 0.1 mg.

**Table 5:** Weather condition and image acquisition schedule during growing period.

DAT	Stage	Weather condition	Image acquisition schedule
1	1	C	B
2		S	B
3		S	A
4		C	A
5	2	C	B
6		C	B
7		S	A
8		C	A
9	3	C	B
10		C	B
11		S	A
12		C	B
13	4	C	A
14		C	B
15		S	A
16		C	B
17	5	C	A
18		C	B
19		C	A
20		S	B

Note: DAT - days after transplanting, S - sunny, C - cloudy; A: image acquisition scheduled from 6:00 h to 18:00 h at 2 h intervals; B: image acquisition scheduled only at 14:00 h.

Images acquired by the imaging system were organized into LemnaBase which is the central database interface for the phenotyping system, and processed through an analysis pipeline specifically adjusted for pakchoi by using LemnaGrid which is an image analysis component of the phenotyping system. Image processing procedure in LemnaGrid consisted of four main steps shown as the flowchart in Supplementary Fig. S3: (1) image preprocessing, extracting target images from LemnaBase; (2) segmentation, separating target plant from the background in the image; (3) feature extraction, analyzing segmentation result and producing phenotypic traits; and (4) post-processing, summarizing feature extraction results of all target images and exporting as “.xls” file.

### Root zone water status discrimination model development

The development of root zone water status discrimination was consisted of three steps: (1) data preprocessing, organizing data for phenotypic analysis and model development; (2) phenotypic traits screening, removing those phenotypic traits with insignificant difference between treatments of root zone moisture level; (3) model development, using different modeling algorithms to classify root zone water status. MetaboAnalyst statistical analysis module (http://www.metaboanalyst.ca) was implemented for data preprocessing and phenotypic traits screening, and R language (windows, R3.2.5, R core Team, 2016) was implemented for model development.

### Data preprocessing

In MetaboAnalyst statistical analysis module, data of extracted phenotypic traits from each image were organized into one datasheet by sample ID (row) and phenotypic traits (column). Empty rows were detected and excluded after data uploaded. Columns with more than 50% empty records were removed. In the case of columns with data missing but less than 50%, the NAs (not available values) were replaced by with a small value (half of the minimum positive values in the original data). All the traits value were normalized by using auto scaling method (mean-centered and divided by the standard deviation of each variable).

### Phenotypic traits screening

Among those phenotypic traits extracted by imaging processing, traits which are insignificantly relevant to treatments may influence the modeling accuracy. In this study, one-way Analysis of Variance (ANOVA) was applied to screen traits resulting an optimal set of explanatory variables for discrimination model development. Traits with P > 0.05 (Fisher’s LSD method) were excluded from the traits set. In addition, the result of ANOVA presented a preliminary overview of the significance of each trait to treatments.

In order to assess the potentiality of using phenotypic traits to develop discrimination model, Partial Least Squares Discriminant Analysis (PLS-DA) was applied, which is a supervised algorithm that uses multivariate regression techniques to predict class membership via linear combination of original variables. The PLS-DA was performed by using plsr function in pls package of R language, and the classification and cross-validation were performed by using the corresponding wrapper function in caret package of R language. The resulted PLS-DA three-dimensional component score plot, which shows the overall data distribution of different treatments, presents the preliminary classification indicating modeling potentiality.

### Discrimination model development

Based on the purpose of multi-classification in this study, three classification modeling algorithms, Random Forests (RF), Neural Network (NN), and Support Vector Machine (SVM) were selected to develop discrimination model and performed with the randomForest, nnet, e1071 packages in R language, respectively.

RF was used as a supervised learning algorithm suitable for the case of high dimensional data analysis. The RF can handle multiple input variables from large databases without variable selection and give estimates of important variables in the classification^25^. RF modeling was performed by using the randomForest package. Ntree (the number of trees) and mtry (the number of features) used to find the best feature were required in RF. In this study the RF model was trained with ntree as 500 and mtry from 2, 5, and 8.

Neural network have been successful as predictive tools in variety domains such as uncovering genotype-phenotype interactions^26^. Researchers reported that properly trained deep neural networks could discover, model, and disentangle latent factors for phenotyping^27^. For NN model in this study, nnet package was used to train the single-hidden-layer feed-forward neural network. There are two main parameters, decay and size, which can be tuned for NN model in R. In this study, the decay were tuned from 0, 0.1, and 0.001, and the size were tuned from 2, 5, and 9.

The goal of a SVM is to create a boundary, called hyperplane, which leads to homogeneous partitions of data on either side. In this study, multiclass SVM model were trained with kernel parameter C (from 10, 100, and 1000) and regularization parameter gamma (from 0.001, 0.01, 0.1, and 0). The best combination of C and gamma leading to the highest prediction accuracy was chosen. For SVM, svm function in e1071 package was used to specify the kernel function, cost, and the gamma function.

The 80% of total samples were selected as the training dastaset via stratified random sampling method for model development and optimization and the rest 20% as the test dataset. In order to prevent the over fitting problems, all three models were trained with the 10-fold cross validation in three repetitions by using the caret package in R language. This method randomly splits the dataset in 10 subsets, among which 9 instances of the data were used to train the model and the other instance was used to validate the training model. By performing three repeats of the 10-fold validation, the average accuracy (percentage of correctly classified samples out of all samples) was used to assess the performance of the model. However, only accuracy is not enough to evaluate the model performance especially for imbalance data or multi class data. Therefore, Cohen’s Kappa coefficient (*k*) and ROC (Receiver operating characteristic) curve were also used to evaluate the model performance. The *k* is a good measure that can handle multi-class well and calculated as:

$${\rm{k}}=\frac{{p}_{o}-{p}_{e}}{1-{p}_{e}}$$

where, p_o_ is the observed agreement, and p_e_ is the expected agreement. The ROC curves of different models were compared according to the area under the curve (AUC) by plotting the true positive rate against the false positive rate. AUC represents the probability that the classifier will assign a higher score to a randomly chosen positive example than to negative example^28^.

## Electronic supplementary material

supplementary information
