# Supplementary material for: Discrimination of plant root zone water status in greenhouse production based on phenotyping and machine learning techniques
Source: Sci Rep. 2017 Aug 15;7:8303. doi: 10.1038/s41598-017-08235-z (PMC5557858; doi:10.1038/s41598-017-08235-z)
Supplement: Supplementary file 1 — supplementary information [file 41598_2017_8235_MOESM1_ESM.doc]

**Discrimination of plant root zone water status in greenhouse production based on phenotyping and machine learning techniques**

Doudou Guo*, Jiaxiang Juan*, Liying Chang, Jingjin Zhang** & Danfeng Huang **

School of agriculture and biology, Shanghai Jiao Tong University, Shanghai, People's Republic of China

*These authors contributed equally to this work.

**Correspondence should be addressed to J.Z. (email: [jj.zhang@sjtu.edu.cn](mailto:jj.zhang@sjtu.edu.cn)) or D.H. (email: [hdf@sjtu.edu.cn](mailto:hdf@sjtu.edu.cn))

Supplementary information


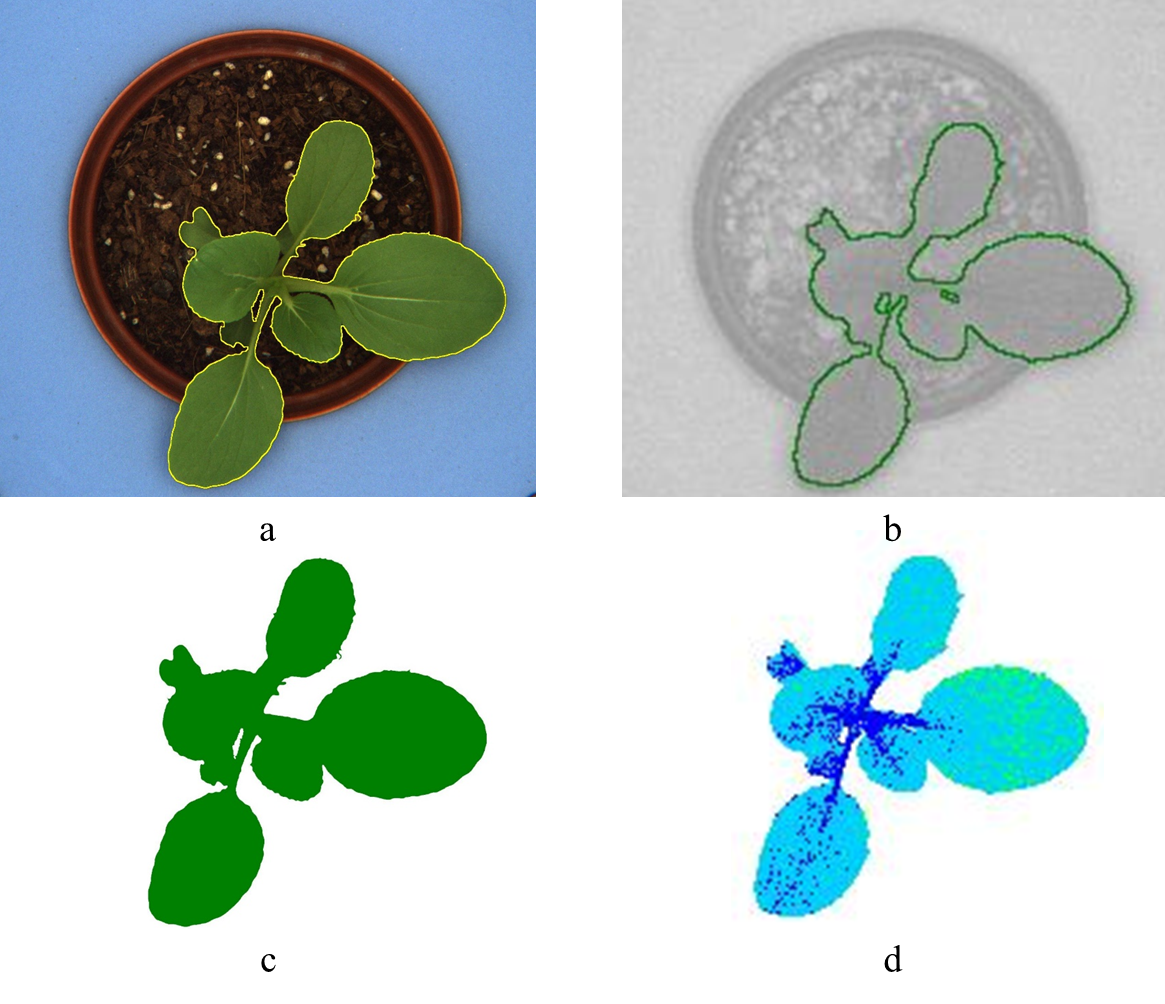


Figure S1. Visible and near-infrared (NIR) images of a plant. (a) visible image, (b) NIR image, (c) plant area extracted from the visible image, and (d) plant area extracted from the NIR image with blue color indicating the NIR intensity


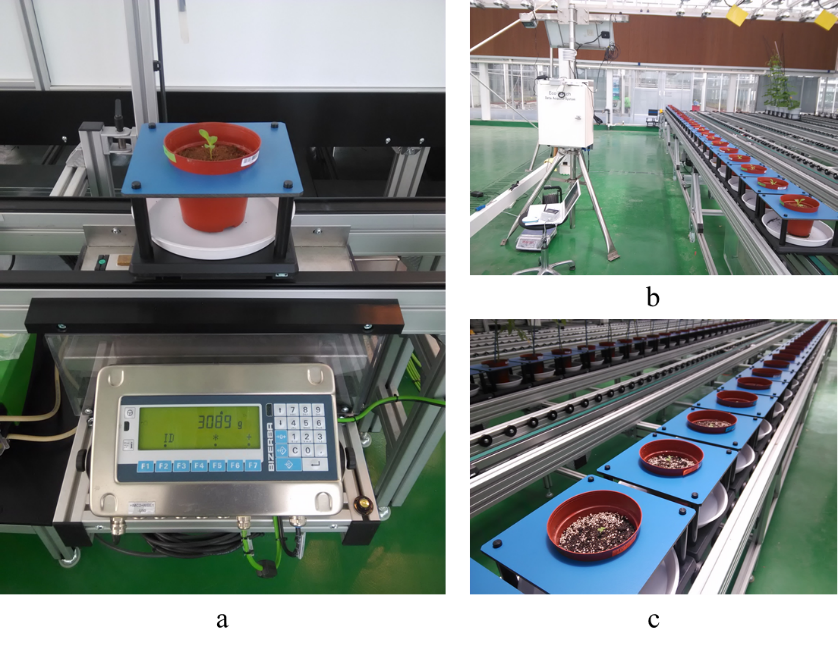


Figure S2. Lemnatec 3D phenotyping system: (a) the sample unit and weighting modeler, (b) growing environment monitoring with PM-11 climate station, and (c) automatic plant conveyors system.


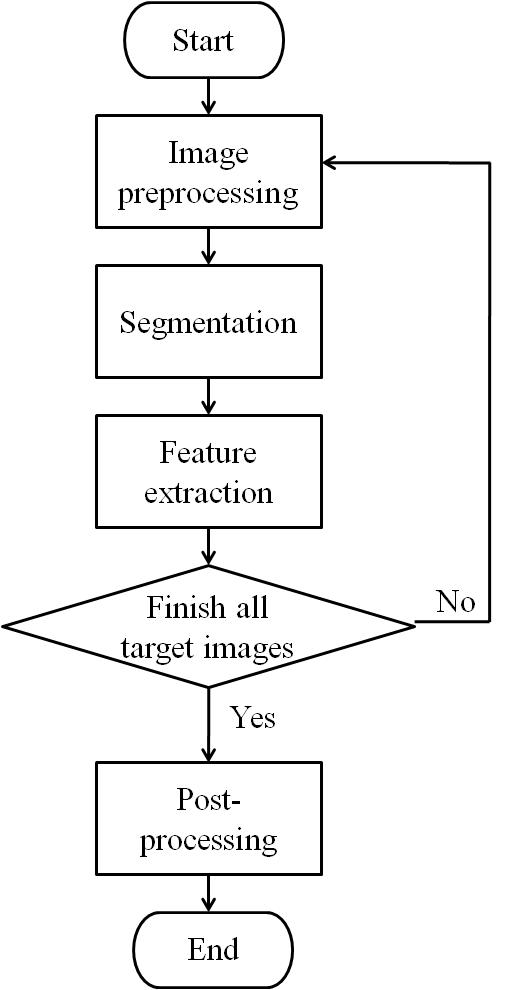


Figure S3. Flowchart of image processing procedure
